# Immune-Mediated Necrotizing Myopathy Associated With Anti-signal Recognition Particle Antibody Complicated With Acute Respiratory Distress Syndrome: A Report of Two Cases

**DOI:** 10.7759/cureus.60477

**Published:** 2024-05-17

**Authors:** Da-Wei Fang, Yi-Min Chen

**Affiliations:** 1 Neurology, Mackay Memorial Hospital, Taipei City, TWN

**Keywords:** acute respiratory distress syndrome, signal recognition particle, srp, immune-mediated necrotizing myopathy, idiopathic inflammatory myopathy

## Abstract

Immune-mediated necrotizing myopathy (IMNM) represents a rare category of inflammatory myopathies characterized by more severe and rapid progression of symmetrical proximal muscle weakness. It is also marked by notably elevated serum muscle enzyme levels and distinct histological features, setting it apart from other types of myositis. Moreover, acute chronic lung respiratory dysfunction is a major comorbidity of great concern. We herein present two cases of IMNM associated with anti-signal recognition particle antibodies complicated by acute respiratory distress syndrome.

## Introduction

Immune-mediated necrotizing myopathy (IMNM) is a rare subclass of idiopathic inflammatory myopathy that manifests with severe disease progression. It is characterized by a notable myofiber necrosis with minimal inflammatory infiltrates, highly elevated creatine kinase (CK) levels, and rare extra-muscular manifestations, distinguishing it from other myopathies such as dermatomyositis or polymyositis [1].

Recent advancements in the understanding of IMNM have been propelled by identifying specific autoantibodies, which have significantly contributed to delineating its pathophysiology and clinical management. These autoantibodies include those against 3-hydroxy-3-methylglutaryl-CoA reductase (HMGCR) and signal recognition particle (SRP), offering insights into the disease's autoimmune underpinnings and aiding in the development of targeted therapies [1,2].

Despite significant progress, the rarity and variability of IMNM present ongoing challenges in understanding its full spectrum of clinical manifestations and responses to treatment. Continuous research efforts are crucial for unveiling the intricacies of IMNM and improving outcomes for those affected by this debilitating condition [2].

## Case presentation

Case 1

We present the case of a 66-year-old female who experienced insidious onset and subacute gradually progressive four-limb weakness accompanied by swallowing difficulty for approximately one month before admission.

The patient had difficulty climbing stairs, standing, wearing clothes, and hanging towers, with no diurnal alternation, fluctuation, specific precipitation, or relieving factors. There were no signs or symptoms of hyperthermia, myalgia, arthralgia, joint deformity, dysarthria, dyspnea on exertion, binocular diplopia, muscle fasciculation, or sensory deficits. Physical examination revealed no Gottron's papules, heliotrope sign, shawl or V-sign, mechanic's hand, or Raynaud's phenomenon. Neurological examination revealed bilateral upper and lower extremity symmetric proximal weakness (Medical Research Council (MRC) scale score=3) with preserved distal muscle power. Serum CK levels were 9719 IU/L.

**Figure 1:**
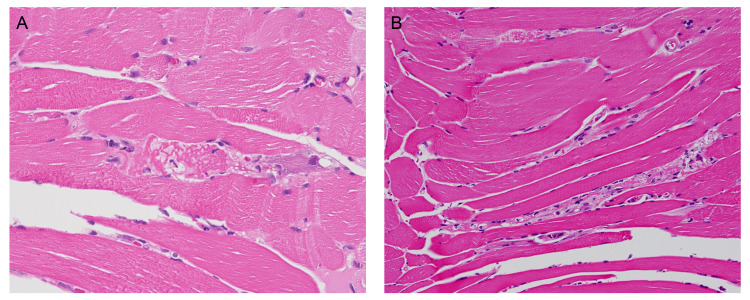
Hematoxylin and eosin staining of muscle biopsies from cases 1 (Figure 1A) and 2 (Figure 1B). Both show (1) A moderate increase in cellularity, suggesting an active pathological process with potential inflammatory or regenerative components. (2) Active necrosis and regeneration of muscle fibers, which is accompanied by degenerative changes in the muscular nuclei and a sparse infiltration of lymphocytes, highlighting ongoing muscle damage and an attempt at repair. (3) Absence of perifascicular atrophy; perifascicular atrophy typically signifies a specific distribution pattern of dermatomyositis. (4) Absence of cytoplasmic inclusions and vacuoles, which may help in differentiating immune-mediated necrotizing myopathy from other specific myopathies such as inclusion body myositis.

Nerve conduction studies revealed bilateral median entrapment neuropathy of the wrist. Electromyography (EMG) revealed positive sharp waves and fibrillation potentials in the right iliopsoas muscle with small amplitude and short-duration motor unit action potentials on volition with early recruitment, suggesting myopathic changes. Because no specific drug history was clarified based on the abovementioned clinical history and examination findings, idiopathic inflammatory myopathy was tentatively diagnosed. Muscle biopsy revealed active necrosis and regeneration with degenerative muscular nuclei and scarcely infiltrating lymphocytes without cytoplasmic inclusions, vacuoles, or significant perifascicular atrophy (Figure 1A).

**Figure 2:**
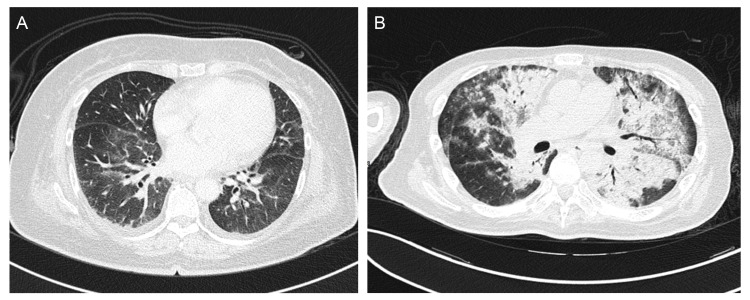
Patient 1’s high-resolution computed tomography (HRCT) images (Figure 2A) show mild reticulations involving bilateral lower lobes, suggestive of interstitial lung disease. Patient 2’s HRCT images (Figure 2B) reveal a diffuse air-space filling process characterized by consolidation mixed with some ground-glass opacities involving both the central lungs, sparing the peripheral parenchyma, and consistent with the butterfly pattern; this is indicative of acute pulmonary alveolar edema.

An investigation of antibodies against autoimmune inflammatory myopathies revealed a high anti-SRP antibody titer. Available metabolic, tumor, and immunological biomarkers tested negative. High-resolution computed tomography (HRCT) revealed mild reticulation involving the lower lobes bilaterally, suggesting interstitial lung disease (Figure 2A).

Therefore, IMNM associated with anti-SRP antibodies was confirmed. The patient received methylprednisolone 1000 mg IV for three days, followed by prednisone 1 mg/kg (50 mg) daily, but with limited benefits. After double-filtration plasmapheresis and rituximab therapy (1000 mg IV, two weeks between each dose) with maintenance azathioprine, the patient partially recovered and was transferred to the rehabilitation department for further management.

**Figure 3:**
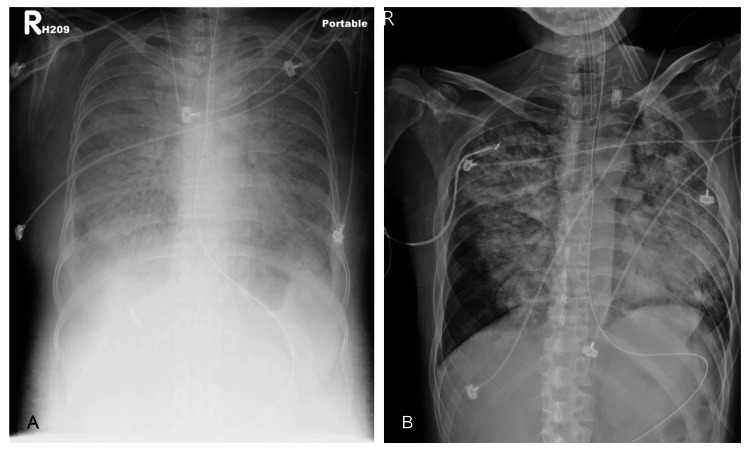
The chest radiographs in cases 1 (Figure 3A) and 2 (Figure 3B) showing severe diffuse bilateral coalescent opacities

The patient tolerated the rehabilitation programs well and was motivated; muscle power recovered significantly (MRC scale ≥4). However, deterioration of dyspnea and easy choking were also reported. A pulmonary function test was performed, which demonstrated that the forced expiratory volume (FEV1)/forced vital capacity (FVC) ratio (84%) was within the normal range, indicating no significant airway obstruction. However, the decreased values of FEV1, FVC (53% and 51% of predicted values, respectively), and total lung capacity (TLC) (47% of predicted values) suggested a pattern of severe restrictive lung disease. Orthopnea, dyspnea with blood-tinged sputum, and chest tightness developed rapidly with respiratory acidosis, and the patient was intubated with mechanical ventilation support and transferred to the intensive care unit. Chest X-ray showed significant infiltration and haziness in both lungs (Figure 3A).

**Figure 4:**
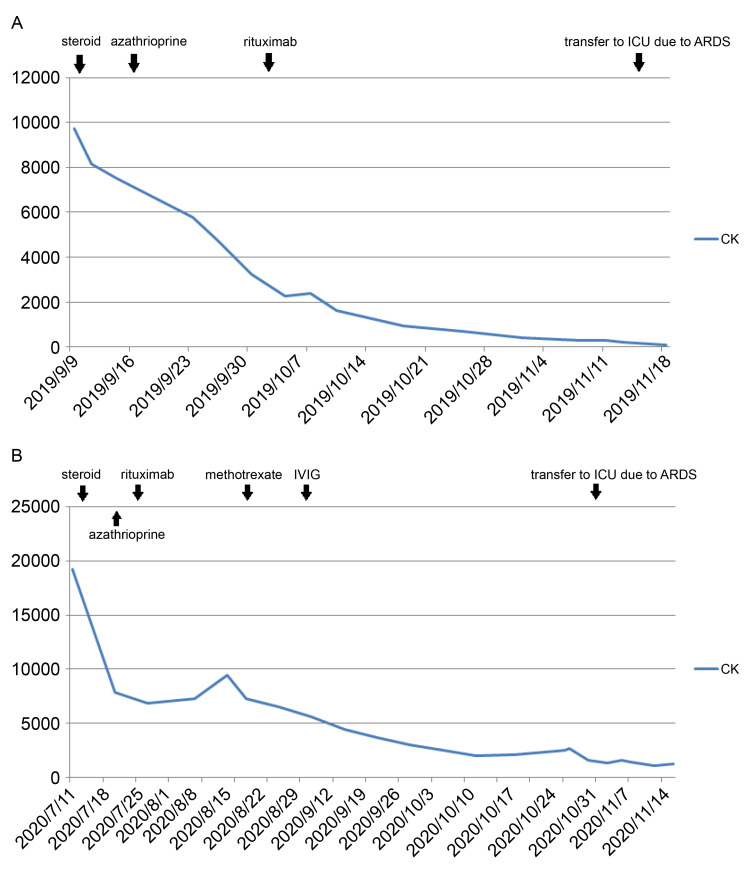
Clinical course of CK levels (IU/L) and medications in cases 1 (Figure 4A) and 2 (Figure 4B) CK: creatinine kinase

No pathogens from blood, urine, and sputum were detected in cultures. During the hospitalization, there were no evident broncho-aspiration incidents; however, the patient experienced some mild choking cough episodes due to swallowing difficulties. Other signs of sepsis identified in this patient included fever, severe hypotension, tachycardia, oliguria, and leukocytosis. We initiated pulse index continuous cardiac output (PiCCO) for hemodynamic monitoring and implemented sedation as part of a lung protection strategy. The patient was positioned prone. However, despite these measures, the lung condition deteriorated, leading to worsening hypoxia and respiratory acidosis. Acute respiratory distress and severe sepsis with septic shock were observed after ICU admission, and the patient died after that due to acute respiratory distress syndrome. The clinical course of CK levels (IU/L) and medications are shown in Figure 4A.

Case 2

The second patient was a 34-year-old male who also suffered from progressive four-limb weakness accompanied by swallowing difficulty for approximately 2 months prior to admission. Neurological examination revealed the symmetric proximal weakness of the bilateral upper and lower extremities (MRC scale score=3) with relatively preserved distal muscle power. The patient denied prior medication use, and no skin rashes were observed. Laboratory tests revealed high levels of CK (19200 IU/L), lactate dehydrogenase (1376 IU/L), and aspartate aminotransferase (AST). Sensory nerve conduction was within normal limits, and motor nerve conduction revealed decreased compound muscle action potentials without significantly slowing conduction velocity. Electromyography (EMG) study revealed spontaneous activity, including fibrillation potentials and positive sharp waves, in all tested muscles and polyphasic short-duration motor unit action potentials with early recruitment.

We checked the paraneoplastic, endocrinological, and infectious profiles; however, no significant findings were observed. Nevertheless, the autoimmune profiles were positive for anti-SRP antibodies. Magnetic resonance imaging revealed linear high T2 signals and enhancement of the visible muscles of the right thigh. Muscle biopsy revealed scattered fiber necrosis with scarce T-lymphocytic infiltrates (Figure 1B). Pulmonary function tests revealed a normal FEV1/FVC (85%) ratio; however, the decreased values of FEV1 (74%), FVC (74%), and TLC (69%) suggested a pattern of mild restrictive lung disease. Under the suspicion of IMNM, pulse steroid therapy (methylprednisolone 1000 mg IV for three days) was administered, followed by double filtration plasmapheresis (every other day for five exchanges). Biochemical data showed reduced CK levels but no remarkable improvement in clinical presentation. Hence, intravenous immunoglobulin and rituximab were administered sequentially; however, no significant recovery from muscle weakness was observed. The patient was discharged from the ward three months later and was kept in rehabilitation.

The patient experienced a rapid onset of shortness of breath and hyperthermia two weeks after discharge, which progressed in the following hours. Therefore, he visited the emergency room. No significant sources of infection were identified during the initial survey period. However, HRCT (Figure 2B) revealed acute pulmonary edema, while serial chest X-ray (Figure 3B) showed progressive bilateral lung infiltration. Subsequently, the patient required intubation for mechanical ventilation support and was later transferred to the ICU. In the ICU, empiric antibiotic and steroid treatments were continued. No pathogens were detected in the sputum or blood cultures. Follow-up chest radiography revealed gradual improvement in pulmonary infiltration and edema. The patient was extubated smoothly and transferred to the general ward. The clinical course of CK levels (IU/L) and medication is shown in Figure 4B.

## Discussion

Two known antibodies are in the IMNM category: anti-SRP and anti-3-hydroxy-3-methylglutaryl-coenzyme A reductase (anti-HMGCR) antibodies. IMNM is distinguishable from dermatomyositis and polymyositis on the pathological features of prominent diffuse myoﬁber necrosis in different stages, with regenerating ﬁbers and absence of signiﬁcant inﬂammatory inﬁltrates [3,4]. Patients with IMNM have muscle weakness and remarkably elevated CK levels as the main clinical manifestations. Patients with IMNM and positive anti-SRP antibodies have more severe muscle symptoms and are more likely to have muscle atrophy, dysphagia, and significantly increased CK levels [5]. Less than 6% of patients with IMNM develop skin rashes such as Gottron's papules, heliotrope signs, shawls, or V-signs [6,7]. Correlation with neoplasms is very rare in the anti-SRP phenotype; however, interstitial lung disease (ILD) should be considered [6]. The overall percentage of ILD in patients with IMNM and anti-SRP antibodies is approximately 17-20% [7-9]. Another report has shown that the incidence of ILD in anti-SRP-positive patients is 64.4% [10]. According to the European Neuromuscular Center treatment guidelines, based on case series and observational cohort studies, rituximab should be used in patients with anti-SRP who fail to respond to steroid pulse therapy and another agent [6,11,12]. To date, there is no consensus regarding the role of plasmapheresis in therapeutic strategies; however, it should be discussed for use in severely affected patients [13].

Both patients in our study with anti-SRP antibodies had acute respiratory distress syndrome. Acute respiratory distress syndrome has been reported in several categories of overlapping autoimmune myositis, including antisynthetase syndrome, and even presented as an initial clinical manifestation [14-16]. In addition to interstitial restrictive lung diseases, acute respiratory distress syndrome is one of the major morbidities of IMNM, which may be attributed to opportunistic infections due to immunosuppressants, dysphagia that leads to aspiration pneumonia, and bilateral diaphragmatic weakness that makes the lungs more vulnerable to infection, all of which may cause acute lung function deterioration in patients with idiopathic inflammatory myositis, especially in the anti-SRP antibody subtype of IMNM, because of the more severe axial muscle involvement and higher risk of interstitial lung diseases [9,17].

Both patients with anti-SRP antibodies developed respiratory failure characterized by rapid widespread significant infiltrates on chest radiography. No specific pathogens were found in the sputum cultures. The adverse effects of interstitial lung disease, acute lung injury, and acute respiratory distress syndrome caused by immunosuppressants such as rituximab and azathioprine are also very rare [18,19], and the onset of respiratory failure was far from the administration of immunosuppressants (Figure 4A, 4B). Hence, the reasons contributing to acute respiratory dysfunction related to anti-SRP antibodies may be the main causes of widespread lung inflammation in both patients.

We suggest that patients with IMNM associated with anti-SRP antibodies should be intensively observed and routinely examined for lung conditions, using respiratory function tests or computed tomography, as well as for distinguishing interstitial lung disease from respiratory muscle weakness. Disease-modifying antirheumatic drugs, especially methotrexate and cyclophosphamide, should be used carefully to avoid drug-induced pneumonitis [18]. In addition to pharmacological management, exercise training, and pulmonary rehabilitation, aggressive management, including ICU care, artificial ventilation, and antibiotics for opportunistic infections, should also be considered.

## Conclusions

For patients diagnosed with IMNM, a tailored and vigilant approach to treatment is paramount. The management strategy for these patients necessitates the judicious selection of disease-modifying drugs that can effectively mitigate the immune response without exacerbating potential side effects. If applicable, the choice of disease-modifying drugs should be informed by a comprehensive understanding of the patient's overall health, disease severity, and response to previous treatments. Given the association of IMNM with ILD in some cases and even acute respiratory distress syndrome in our rare cases, rigorous monitoring of pulmonary function is critical. This includes assessments through respiratory function tests, which can provide early detection of declining lung function and enable timely intervention. Additionally, computed tomography scans of the chest are invaluable for identifying subtle changes in lung tissue that may not be apparent in clinical examination or standard radiographs.

The integration of these diagnostic tools, alongside a multidisciplinary approach involving rheumatologists, neurologists, and pulmonologists, ensures a comprehensive care plan tailored to the patient's individual needs. This approach aims to control the myopathy and preemptively address potential complications, thereby improving the quality of life and prognosis for those affected by anti-SRP antibody-associated IMNM. Close collaboration between the patient and the healthcare team and patient education about recognizing signs of worsening lung function is essential for successfully managing this condition.
